# Chromatin Morphology in Human Germinal Vesicle Oocytes and Their Competence to Mature in Stimulated Cycles

**DOI:** 10.3390/cells12151976

**Published:** 2023-07-31

**Authors:** Daniil Salimov, Tatiana Lisovskaya, Junko Otsuki, Alexandre Gzgzyan, Irina Bogolyubova, Dmitry Bogolyubov

**Affiliations:** 1 Clinical Institute of Reproductive Medicine, Yekaterinburg 620014, Russia; tv.lis@mail.ru; 2 Assisted Reproductive Technology Center, Graduate School of Environmental, Life, Natural Science and Technology, Okayama University, Okayama 700-8530, Japan; otsuki.midori.junko@gmail.com; 3 Research Institute of Obstetrics, Gynecology and Reproductology Named after D. O. Ott, St. Petersburg 199034, Russia; agzgzyan@gmail.com; 4 Institute of Cytology, Russian Academy of Sciences, St. Petersburg 194064, Russia; ibogol@mail.ru; 5 Department of Histology and Embryology Named after Prof. A.G. Knorre, St. Petersburg State Pediatric Medical University, St. Petersburg 194100, Russia

**Keywords:** human oocyte, germinal vesicle, spontaneous maturation, stimulated cycle, aneuploidy rate, atypical nucleolus, chromatin configuration, karyosphere

## Abstract

The search for simple morphological predictors of oocyte quality is an important task for assisted reproduction technologies (ARTs). One such predictor may be the morphology of the oocyte nucleus, called the germinal vesicle (GV), including the level of chromatin aggregation around the atypical nucleolus (ANu)—a peculiar nuclear organelle, formerly referred to as the nucleolus-like body. A prospective cohort study allowed distinguishing three classes of GV oocytes among 135 oocytes retrieved from 64 patients: with a non-surrounded ANu and rare chromatin blocks in the nucleoplasm (Class A), with a complete peri-ANu heterochromatic rim assembling all chromatin (Class C), and intermediate variants (Class B). Comparison of the chromatin state and the ability of oocytes to complete meiosis allowed us to conclude that Class B and C oocytes are more capable of resuming meiosis in vitro and completing the first meiotic division, while Class A oocytes can resume maturation but often stop their development either at metaphase I (MI arrest) or before the onset of GV breakdown (GVBD arrest). In addition, oocytes with a low chromatin condensation demonstrated a high level of aneuploidy during the resumption of meiosis. Considering that the degree of chromatin condensation/compaction can be determined in vivo under a light microscope, this characteristic of the GV can be considered a promising criterion for selecting the best-quality GV oocytes in IVM rescue programs.

## 1. Introduction

The process of oocyte development includes two-stage meiotic division, resulting in the formation of haploid female gametes ready for fertilization and further zygote development [1]. The diplotene stage of meiotic prophase—at which the oocyte nucleus is called the germinal vesicle (GV) [2]—is crucial for the oocyte to transit from growth to maturation. Oocyte maturation is a complex process in which cytoplasmic and nuclear factors contribute [3]. After the GV stage and the germinal vesicle breakdown (GVBD), two meiotic stages are the key waystations of oocyte maturation— metaphase I (MI) accompanied by the emission of the first polar body (PB1) and metaphase II (MII) determined by the release of the second polar body (PB2). Each stage is equally important for the oocyte as it allows the female gamete to attain an optimal state of developmental competence [4]. However, it is at the GV stage that the oocyte primarily acquires the capacity to be fertilized and further develops properly into a future embryo [5]. Both nuclear and cytoplasmic factors and structures substantially involved in oocyte maturation should be created, accumulated, and integrated into further developmental events [6]. Some determinants affecting meiotic progression have already been elucidated, while other factors associated with the developmental arrest at the GV stage have yet to be defined [7,8].

The GV is characterized by global chromatin rearrangements at both morphological and molecular levels [9]. In some mammals, including humans, chromatin rearrangements within the GV coincide with the transformation of the nucleolus into a transcriptionally inert nuclear body called the nucleolus-like body (NLB) or, more recently, the atypical nucleolus (ANu) [10]. In this case, the condensed chromatin forms at the ANu periphery a more or less compact perinucleolar ring, sometimes called a karyosphere [11,12].

There is no universal classification of chromatin configuration in human GV oocytes. The main architectural patterns of chromatin found in human GVs are the following [13,14]: (i) no karyosphere (peri-ANu ring) is observed, chromatin is dispersed through the GV, and only some heterochromatin blocks are located both in the vicinity of the ANu and outside; (ii) the ANu is incompletely rimmed by chromatin; (iii) the ANu is completely surrounded by chromatin, but some chromatin masses are scattered outside the peri-ANu rim; (iv) all chromatin surrounds the ANu in the GV, forming a compact well-formed karyosphere. Approximately 50% of human oocytes retrieved from antral follicles have rather condensed chromatin [15].

During the GVBD and the emission of the PB1 and PB2, the processes of repositioning and remodeling of organelles occur, including mitochondria, the Golgi complex, and the endoplasmic reticulum [6,16]. This has been vividly demonstrated using a modern time-lapse technique [17]. The functional significance of such rearrangements during oocyte maturation is not completely clear. It is assumed that changes in the oocyte structure play a role in the maturation and further development of the embryo. For example, the central position of the GV in both mouse [18] and human oocytes [19] correlates positively with the tendency of the oocyte to resume meiosis and depends on the functional state of the cytoskeleton [20].

Currently, there is no unified method for assessing the initial quality and development potential of GV oocytes and subsequent embryos. However, such an integrated approach would be useful to course the prospects for infertility treatments. Along with the morphological parameters of mature oocytes at the metaphase II (MII) stage, some changes in the parameters of GV oocytes—e.g., a number of immature oocytes—can probably serve as a criterion for assessing the quality of response to hormonal stimulation [21].

A fully developed GV oocyte is capable of spontaneous maturation to MII when isolated from follicles >3 mm [22]. The levels of implantation and quantity/quality of blastocysts for in vitro matured oocytes, both in the stimulated cycle (GV rescue) [23] and classical non-stimulated IVM [24,25], were found to be lower compared to a conventional IVF cycle. The low percentage of oocytes matured in vitro and the poor quality of further embryos indicate that a significant proportion of oocytes retrieved from antral follicles are meiotically incompetent.

It is likely that GV oocytes with more compacted chromatin are more capable of developing. For example, porcine oocytes with a fully developed karyosphere are better suited for IVF after in vitro maturation [26]. Contrariwise, mouse GV oocytes with predominantly diffuse chromatin can fertilize, but the descendant embryos cannot reach the blastocyst stage in vitro [27]. Moreover, human oocytes with a similar chromatin pattern show some ultrastructural signs of cytoplasmic degeneration [28]. It has been experimentally shown that chromatin remodeling defects in oogenesis also cause aneuploidies, leading to pre- and post-implantation developmental arrest, implantation disorders, and spontaneous abortions [29]. Such widely-used morphological and morphometric indicators as the state of the nucleoplasm, the position of the GV, the shape of the nuclear envelope, and the size of the oocyte are associated with chromatin condensation [30]—a potential marker of oocyte quality in IVM cycles.

Here, we performed a prospective cohort study of GV oocytes retrieved from patients treated for infertility with IVF. The aim of this study was to reveal whether the morphology of the chromatin compartment correlates with the capability of GV oocytes to resume meiosis.

## 2. Materials and Methods

### 2.1. Study Design

The study was conducted at the Clinical Institute of Reproductive Medicine (Yekaterinburg, Russia) between 2021 and 2022, where the actual work with patients and GV oocytes was carried out from May to August 2021. Patients applied to the clinic for infertility treatment using ARTs. The infertility factors in the analyzed IVF treatments are presented in Supplement (Figure S1). The average clinical parameters of the patients whose oocytes were retrieved are shown in Table S1. All women were of Caucasian (Eurasian) race, various ethnic groups inhabiting the Russian Federation, as well as various social strata without restrictions. For patients, there were no restrictions on age or the level of AMH and FSH. Inclusion parameters were the presence of own (autologous) oocytes after a puncture, the appointment of ICSI by the attending physician, and the presence of one or more GV oocytes after denuding. The material of GV oocytes used in this study was intended for disposal and not for use in IVF programs. The study used all retrieved GV oocytes not intended for ART.

**Figure 1:**
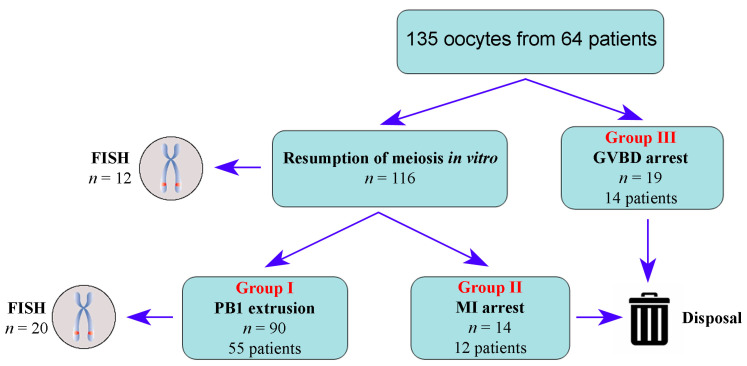
A diagram illustrating the studied groups of human oocytes.

One hundred and thirty-five oocytes retrieved from sixty-four patients were included in this study. Each oocyte was perceived as a case. All oocytes were divided into three groups: Group I (*n* = 90 from 55 patients)—GV oocytes that spontaneously resumed meiosis in vitro and completed the first meiotic division; Group II (*n* = 14 from 12 patients)—GV oocytes that resumed maturation but stopped developing at metaphase I (MI arrest); Group III (*n* = 19 from 14 patients)—GV oocytes that stopped before the onset of GV breakdown (GVBD arrest). Oocytes from the same patient may fall into different groups. Thirty-two randomly selected oocytes of the Groups I and II (MI and MII) were used for FISH (Figure 1).

### 2.2. Ovarian Stimulation, Oocyte Retrieval, and In Vitro Culture

All patients had a history taken prior to ovarian stimulation. The level of anti-Müllerian hormone (AMH) was measured in venous blood using a miniVIDAS device (Biomerieux, Craponne, France); the levels of follicle-stimulating hormone (FSH) and thyroid-stimulating hormone (TSH) were measured using an Access device (Beckman Coulter, Inc., Brea, CA, USA) to the manufacturer’s recommendations.

Patients were stimulated with gonadotropin-releasing hormone (GnRH) agonist and antagonist protocols. Total dose of gonadotropins were ≥1500 IU. When three or more dominant follicles reached 17–18 mm, patients were administered with ovulation triggers: human chorionic gonadotropin (hCG) (Ovitrelle, Merck Serono, Darmstadt, Germany; Pregnyl, Organon Jersey City, NJ, USA) or, if there were 15 or more dominant follicles, GnRH agonist (Decapeptyl, Ferring Pharmaceuticals, Saint-Prex, Switzerland; Diphereline, Ipsen Pharma, Paris, France).

Transvaginal follicle puncture was performed using 17 G aspiration needles 36 h after trigger injection, under intravenous anesthesia (Propofol), using a vaginal transducer with a U/S control. The material was collected in 5 mL syringes. Each follicle was washed separately in 2 mL of Modified HTF medium with HEPES (FUJIFILM Irvine Scientific, Santa Ana, CA, USA). During puncture, before aspiration of the contents, follicles were measured and ranked according to their diameter: large (≥18 mm), medium (16–17 mm), and small (≤15 mm) [31]; the retrieved cumulus-oocyte complexes were placed in separate marked dishes. Oocytes were removed from the cumulus-oocyte complexes under a microscope in a laminar box with a built-in heating surface under aseptic conditions at 37 °C, washed from blood and follicular fluid in a MHM culture medium (Multipurpose Handling Medium Complete, FUJIFILM Irvine Scientific, Santa Ana, CA, USA) for working with gametes and embryos outside the incubator and then placed in human tubal fluid complete medium (HTF) (FUJIFILM Irvine Scientific, Santa Ana, CA, USA) in a CO_2_ incubator (6.0% CO_2_, 37 °C) for 2 h before further manipulations.

After 2 h of preincubation in HTF medium, oocytes were denuded using sterile capillaries (140 and 170 μm in diameter) by pipetting in 80 IU/mL hyaluronidase (Irvine, USA) in accordance with the recommendations of the culture media manufacturer. After three-fold washing in MHM, the stage of oocyte development was assessed under a Nikon Ti microscope (Nikon Instruments, Tokyo, Japan) equipped with a relief contrast system. Oocytes accompanied by one PB were evaluated as stage MII oocytes [32] and further used for fertilization according to the patient’s treatment protocol. Oocytes without a PB and with an intact nucleus (GV) were used for further scientific purposes in this study. GV oocytes in a HTF medium without growth factors were placed in a CO_2_ incubator in culture dishes with microwells (EmbryoScope, Vitrolife, Göteborg, Sweden), and time-lapse microscopy (1 frame/10 min) was performed for the subsequent 48 h.

### 2.3. Morphological and Morphometric Assessment

**Figure 2:**
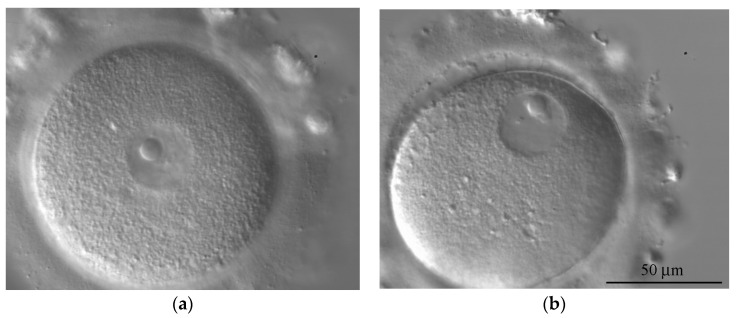
Human oocytes with central (**a**) and peripheral position (**b**) of the GV.

The following morphological parameters were assessed: the central or peripheral position of the GV [18,33] (Figure 2) and the level of chromatin condensation/aggregation on the ANu surface [13,14]. According to the degree of chromatin condensation, oocytes were divided into three classes—A, B, and C (Table 1).

**Figure A1:**
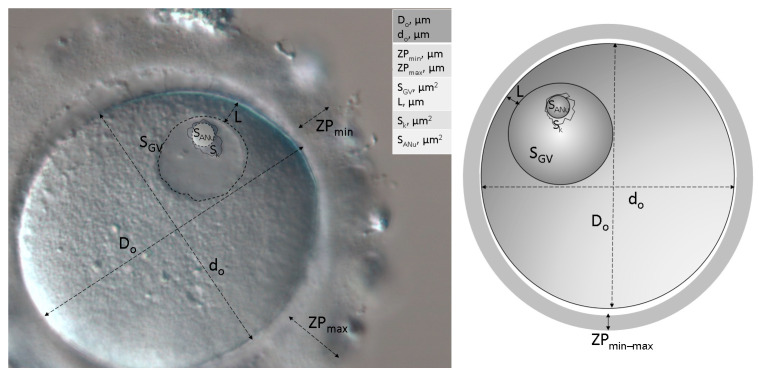
Main morphometric parameters measured in the study: long diameter (D_o_, μm); short diameter of the oocyte (d_o_); minimum and maximum thickness of *zona pellucida* (ZP_min_/ZP_max_); eccentricity of the nucleus (L); projection areas of the nucleus (S_GV_), atypical nucleolus (S_ANu_), and karyosphere (S_k_).

Oocyte morphometry was performed in glass-bottom dishes (WilCoDish, WillCo Wells B.V., Amsterdam, The Netherlands) 38 h after the trigger of ovulation administration and immediately after oocyte denuding, with the use of an inverted microscope Nikon Eclipse Ti-U, T-P2 polariser (Nikon Instruments, Tokyo, Japan), equipped with a 40×/0.60 DIC Plan Fluor objective. Oocytes were captured three times using a cytometric software package (OCTAX EyeWare Imaging Software, v. 2.2.3.327, Vitrolife, Göteborg, Sweden). The following morphometric parameters were measured: long diameter (D_o_, μm); short diameter of the oocyte (d_o_, μm); minimum and maximum thickness of *zona pellucida* (ZP_min_/ZP_max_, μm) (Figure A1—Appendix A).

The oocyte projection area (S_o_, μm^2^) was calculated as the ellipse area using the formula:

S_o_ = π × D_o_ × d_o_/4 (1)

The average oocyte diameter (D_av_, μm) was calculated as the arithmetic mean between the D_o_ and d_o_ values. The oocyte radius (R_o_, μm) was calculated using the formula:

R_o_ = D_av_/2 (2)

Morphometric measurements of GVs and ANu–chromatin complexes (karyospheres) were performed using JMicroVision v. 1.2.7 software tool (Geneva, Switzerland). Calibration of the software product was carried out using a reference object (1-mm scale, Nikon Instruments, Tokyo, Japan). The following morphometric parameters were measured: the projection area of the nucleus (S_GV_, μm^2^); the karyosphere projection area (S_k_, μm^2^); the ANu projection area (S_ANu_, μm^2^); the minimum distance from the nuclear envelope to the oolemma (L, µm). Several additional parameters, including the diameter of the nucleus (D_GV_, µm), the radius of the nucleus (R_GV_, µm), relative eccentricity (L_ecc_, %), and chromatin projection area (S_ch_, μm^2^), were calculated as follows:

D_GV_ = 2 × √S_GV_/π, (3)

R_GV_ = √S_GV_/π, (4)

L_ecc_ = 100 × (R_o_ − R_GV_ − L)/(R_o_ − R_GV_), (5)

S_ch_ = S_k_ − S_ANu_ (6)

### 2.4. Morphodynamics

The morphometry of the chromatin-ANu complex was carried out at the following intervals after the trigger of ovulation: 38 h (immediately after denuding), 44 h (i.e., 6 h after denuding), and 56 h (i.e., 18 h after denuding). To assess the oocyte morphodynamical points during the maturation, the following indicators were evaluated: onset of GVBD (T_GVBD_) and extrusion of the PB1 (T_PB1ex_, min).

### 2.5. Biopsy and Fluorescent In Situ Hybridization (FISH)

**Figure A2:**
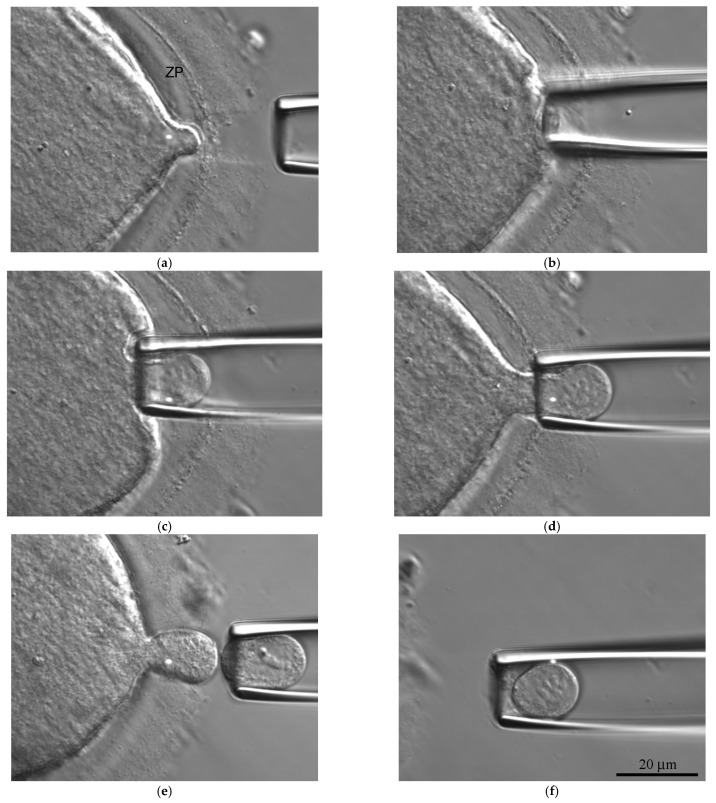
Consecutive steps of metaphase plate biopsy. (**a**) Laser dissection of *zona pellucida* (ZP); (**b**) penetration of the capillary; note the tight contact with the area containing the spindle; (**c**) suction of the karyoplast; (**d**) extraction of the karyoplast through ZP; (**e**) detaching the karyoplast from the remaining ooplasm; (**f**) transfer of the karyoplast.

To evaluate aneuploidy rates in immature oocytes, we performed a biopsy of the karyoplasts containing the first meiotic spindle (MI). The steps of the biopsy procedure are illustrated in Figure A2 (Appendix A). Then, to assess aneuploidy rates in mature oocytes (MII), we performed a simultaneous biopsy of the karyoplasts containing the second meiotic spindle and the PB1. Biopsies were performed with 15-µm micropipettes in a mHTF culture medium buffered with HEPES containing 10% HSA and 5 µg/mL cytochalasin B (Sigma-Aldrich, Burlington, MA, USA; Merck Serono, Darmstadt, Germany) as an actin polymerization inhibitor. A laser system (OCTAX, Vitrolife, Göteborg, Sweden) was used for assisted dissection of the ZP. DIC optics was used for imaging.

Fixation of the karyoplasts and PBs was carried out on glass slides under a phase contrast microscope. The karyoplasts and PB1 were briefly placed in a hypotonic solution (0.6% BSA and 1% sodium citrate, Sigma-Aldrich, Burlington, MA, USA; Merck Serono, Darmstadt, Germany), then transferred on a glass slide in a minimum volume of the solution. After complete drying, one to two small drops of fixing solution (1:3 mixture of acetic acid and methanol, Sigma, Burlington, MA, USA; Merck Serono, Darmstadt, Germany) were applied. The Vysis MultiVysion PB Multi-color Probe kit (Abbott, Abbott Park, IL, USA) was used to identify common aneuploidies (Table 2). The number of copies of chromosomes was estimated by the number of FISH fluorescent signals using a Leica DM4000B fluorescent microscope (Leica Microsystems, Wetzlar, Germany) equipped with red (600–690 nm), green (500–580 nm), aqua (400–440 nm), blue (420–500 nm), and gold (540–550 nm) filters (Chroma Technology Corporation, Bellows Falls, VT, USA), and a 100× objective.

Fluorescent signals were counted in the MI spindle or jointly in the PB1 and the MII spindle (PB1/MII). In MI, the number of fluorescent signals in each chromosome equal to 4 was taken as normal; in PB1/MII, the number of fluorescent signals 2/2 was also taken as normal. The variants 3/1, 1/3, 0/4, and 4/0 were considered chromosome segregation errors. Fluorescent signal variants in MI equal to 0 or 2 were taken as a chromosome aggregation error (loss of chromosomes). Fluorescent signal variants 1/1, 2/0, 0/2, and 0/0 in PB1/MII were suggested as a chromosome aggregation error (loss of chromosomes). If segregation errors were detected for some chromosomes and aggregation errors for others, then a conclusion was made about a combined error.

### 2.6. Statistical Analysis

Qualitative features were described by simply indicating the number of patients and the proportion (in percent) for each category. The statistical significance of differences between these features was assessed using the χ^2^ test with Yates correction and Fisher’s exact test. All quantitative parameters were tested for normal distribution using the Shapiro–Wilk test. Taking into account that the distribution of some quantitative parameters was significantly different from the normal one, statistical analysis was carried out using nonparametric criteria. The values are described as a median and boundaries of the interquartile interval (25–75%). The statistical significance was assessed using the Mann–Whitney test. The differences were considered statistically significant at *p* < 0.05 in all cases.

## 3. Results

### 3.1. Anamnestic Predictors

A comparative analysis of the ability of GV oocytes to resume maturation and develop to the MII stage was performed, taking into account the collected history of patients. All oocytes were divided into three groups according to their ability to resume meiosis (Figure 1). Anamnestic data for different groups of oocytes are summarized in Table 3.

Patients whose oocytes cease to develop at stages MI and GV (Groups II and III, respectively) significantly differ in age. There are also significant differences between Groups I and III in the AMH level in the blood, which reflects differences between the capacity of oocytes to complete a full cycle of maturation, including resumption and completion of meiosis to MII and the tendency of oocytes to stop development at the GV stage (Table 3).

Significant differences are found in the competence of oocytes to initiate meiosis and extrude the PB1 in the following groups of patients: (i) with polycystic ovary syndrome (PCOS), more common in Group II than in Group I; (ii) with endometriosis, more common in Group III than in Group I; and (iii) after ovarian resection, which was significantly less common in Group III with GV-arrested oocytes compared with Group I with complete meiosis. While ovarian resection in Group I is a fairly common event, no cases were noted in Group III. Among the compared groups of patients, there are no significant differences in body mass index (BMI), the number of antral follicles, FSH and TSH levels, type of infertility (primary or secondary), and premature ovarian failure (POF) (Table 3).

To assess the effect of hormonal stimulation on the ability of oocytes to mature spontaneously, we evaluated the stimulation protocols and ovulation triggers, as well as the drugs purchased from different manufacturers, which were used in different groups of patients (Table 4). In Group I, a short gonadotropin-releasing hormone (GnRH) antagonist protocol was used more frequently, but the differences were not significant. No differences were found between Groups I/II and Group III when human chorionic gonadotropin (hCG) or GnRH agonists were used as triggers, either. Further, no significant differences were found in patients treated with the same drug supplied by different manufacturers.

To assess the adequacy of the ovarian response to hormonal stimulation, we monitored the estradiol level (E_2_) in the blood on the day of transvaginal aspiration (Table 5). In other words, we tested how actively the ovaries respond to the growth of dominant follicles in FSH-stimulated patients. Despite a wide range observed, no significant differences are found. Other characteristics of clinical response to stimulation are also presented in Table 3. No significant differences are detected in the number of dominant follicles per puncture, the number of received oocytes, the number of GV and mature oocytes, the quality of the embryos received on days three and five in cycles, and clinical pregnancy rates (CPR).

### 3.2. Morphological Predictors of GV Oocyte Maturation

**Figure 3:**
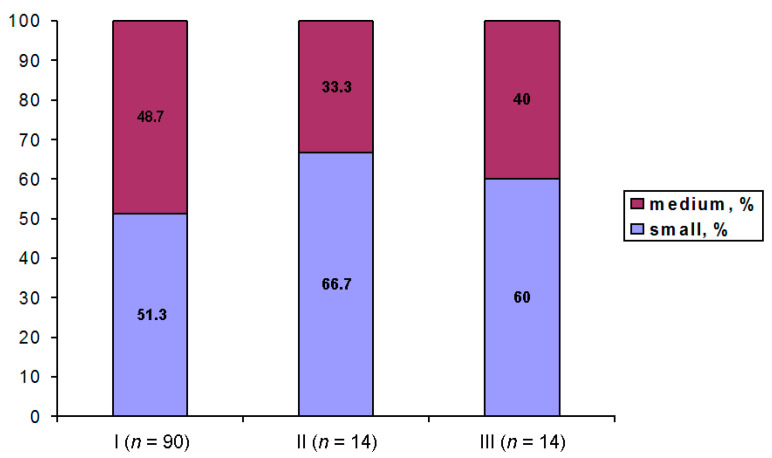
Percentage of GV oocytes retrieved from small and medium follicles in Groups I, II, and III that differ by their competence to resume meiosis.

All GV oocytes were retrieved from the medium (16–17 mm) and small (≤15 mm) follicles. Larger follicles that contain mature oocytes (MI and MII) were not used in this study. Follicle size does not differ significantly in different groups/subgroups of patients. The percentage of GV oocytes retrieved, respectively, from small and medium follicles is shown in Figure 3.

**Figure 4:**
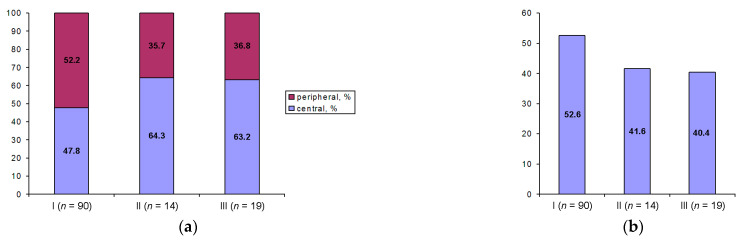
Percentage of oocytes with peripheral and central localization of the GV (**a**) and the relative displacement of the GV (L_ecc_, %) (**b**) in oocyte groups differing by their competence to resume meiosis (I, II, and III).

The representative oocytes with central and peripheral positions of the GV are shown in Figure 2. Although the central position of the GV was somewhat more common in Groups II and III, the studied groups do not differ significantly by this parameter, regardless of whether the qualitative assessment of the GV (central or peripheral) was performed or the relative displacement of the GV (L_ecc_) was calculated as described in Section 2 (Figure 4a,b). 

Large-scale morphometric data are presented in Table 6. In all groups, GV oocytes did not differ significantly in size. However, Groups II and III showed little difference in the length of the oocyte long axis (D_o_), but the calculated average oocyte diameter (D_av_) and oocyte projection area (S_o_) do not differ significantly. In all the studied groups, there were no significant differences in the thickness of *z. pellucida* (ZP_min_/ZP_max_). The nucleus projection area (S_GV_) and the calculated diameter of the nucleus (D_GV_) were larger in Group III; the differences are significant between Groups I and III but not between Groups II and III.

### 3.3. Dynamics of GV Oocyte Maturation

Using time-lapse microscopy, we estimated the time until the onset of GVBD (T_GVBD_). This period was longer in Group II than in Group I (Table 7). Among all GV oocytes that entered and finished maturation, the time from the onset of GVBD to the emission of the PB1 (T_PB1ex_) was approximately 16.5 h (995.0 min), and time was scattered minimally in this case.

### 3.4. Configuration of Chromatin

**Figure 5:**
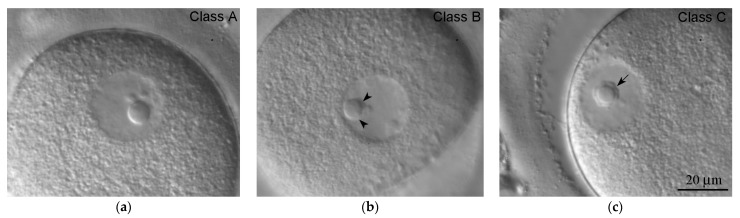
Representative human GV oocytes of three classes (A, B, and C) that differ in chromatin configuration and its association with the atypical nucleolus (asterisks) viewed using DIC optics (**a**–**c**). In Class A oocytes (**a**), chromatin is predominantly distributed throughout the GV; only rare blocks of heterochromatin are visible. In Class C oocytes (**c**), all the chromatin is assembled around the atypical nucleolus, forming a ring (arrow). Class B oocytes (**b**) exhibit all intermediate chromatin configurations; gaps in the incomplete ring around the atypical nucleolus are shown (arrowheads).

**Figure 6:**
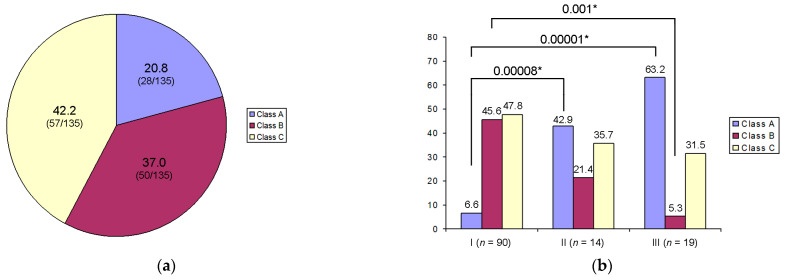
Percentage of oocytes with different chromatin configurations (Classes A, B, C): (**a**) of all oocytes retrieved; (**b**) in Groups I, II, and III that differ by their competence to resume meiosis; asterisks indicate significant differences and corresponding *p*-values.

The chromatin configuration in GV oocytes has attracted special attention in this study. Using DIC microscopy, we were able to differentiate three main types of chromatin configuration, referred to as Classes A, B, and C (Figure 5 and Table 1). There was no case when the projection area of chromatin aggregated on the ANu (S_ch_) was equal to zero µm, indicating that the retrieved dominant follicles contained oocytes with different levels of heterochromatin aggregation at the ANu surface. Although at least one prominent heterochromatin block was always seen adjacent to the ANu, we observed different chromatin patterns, which are the closest to those observed in Classes A, B, and C oocytes according to the classification proposed by Miyara et al. [14], see also Table 1. At the same time, we were unable to distinguish chromatin masses in the nucleoplasm from the “threads of dispersed chromatin” characteristic for Class D oocytes, according to Combelles et al. [13]. In our material, Class C oocytes were the most common, while Class A oocytes were the rarest (Figure 6a).

The percentage of oocytes with different degrees of chromatin compaction is shown in Figure 6b. Class B and C oocytes were more frequently detected among the oocytes capable of complete maturation. The oocytes that entered maturation but were arrested at the MI stage more often belonged to Class A. The oocytes that did not enter maturation were more often classified as Class A. At the same time, the comparison groups did not show significant differences concerning the Class C oocytes. Contrarily, oocytes significantly differ in Groups I and III by the Class B chromatin configuration. Finally, there are significant differences between Groups I and III and between Groups I and II by the presence of Class A oocytes, but Groups II and III do not differ significantly (Figure 6b).

**Figure 7:**
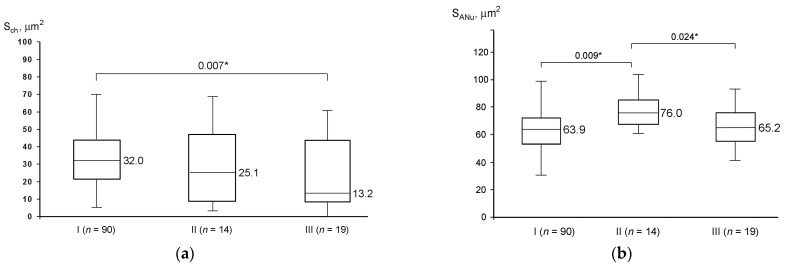
Aggregation level of condensed chromatin, S_ch_ (**a**), and atypical nucleolus size, S_ANu_ (**b**), in oocyte groups differing by their competence to resume meiosis (I, II, and III); asterisks indicate significant differences and corresponding *p*-values.

The parameter S_ch_ showed some differences in the studied groups of oocytes, demonstrating a significantly reduced S_ch_ in Group III compared with Group I (Figure 7a). The ANu projection area (S_ANu_) is significantly larger in the oocytes of Group II than in the oocytes of Groups I and III (Figure 7b).

**Figure 8:**
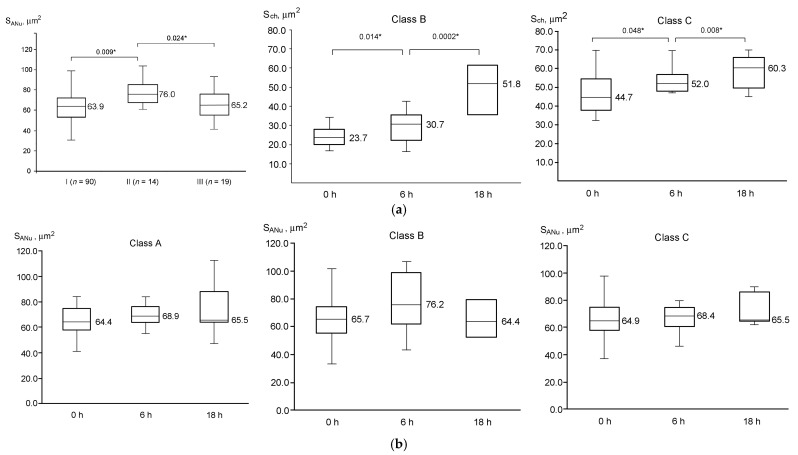
Dynamics of projection areas of chromatin, S_ch_ (**a**), and atypical nucleolus, S_ANu_ (**b**), during in vitro development of GV oocytes with different chromatin configurations (Classes A, B, C); asterisks indicate significant differences and corresponding *p*-values.

The level of chromatin aggregation on the ANu surface changes during in vitro culture of GV oocytes in classes B and C. The parameter S_ch_ noticeably increased in these oocytes already after 6 h of cultivation, expanding even more by 18 h (Figure 8a). In Class A oocytes, S_ch_ also tends to increase, but the differences are not significant (Figure 6a). The ANu projection area (S_ANu_) is not influenced significantly during the cultivation of GV oocytes in vitro (Figure 8b).

### 3.5. Aneuploidies in MI and MII Oocytes

**Figure 9:**
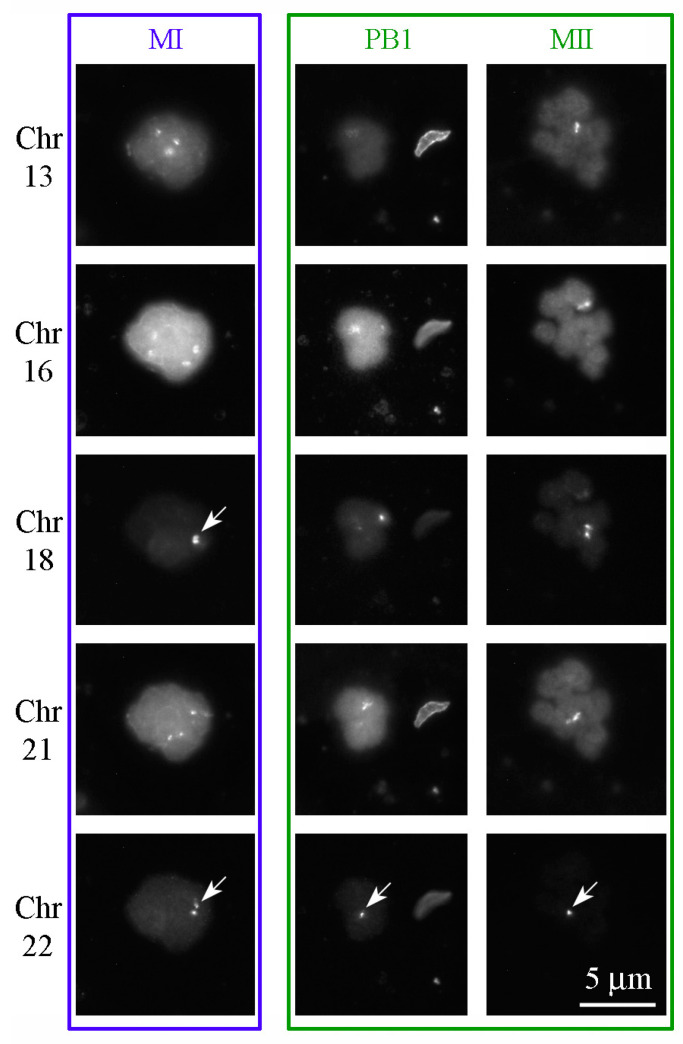
FISH to reveal aneuploidy for chromosomes (Chr) 13, 16, 18, 21, and 22 in an oocyte at metaphase I (MI, left column, framed blue) and an oocyte at metaphase II (MII, right column) with the first polar body (PB1, central column) (framed green). Aneuploidies are indicated by arrows: two—instead of four—copies of Chr 18 and 22 in MI and one—instead of two—copies of Chr 22 in PB1 and MII. Both examples are derived from Class A GV oocytes.

Since we confirmed the possibility of GVBD and MI spindle formation in oocytes with an incomplete karyosphere (Class A), we assumed that, in this case, an incomplete spindle is formed. Such a spindle, which apparently does not unite all chromosomes, leads to the loss of chromosomes and pre-segregation aneuploidy. To investigate this, we dissected ZP with a laser, after which the karyoplasts containing MI and a small amount of ooplasm as well as the karyoplasts containing MII together with PB1 were isolated (Figure A2—Appendix A), and then stained with locus-specific (LSI) and centromeric (CEP) fluorescent probes (Figure 9).

**Figure 10:**
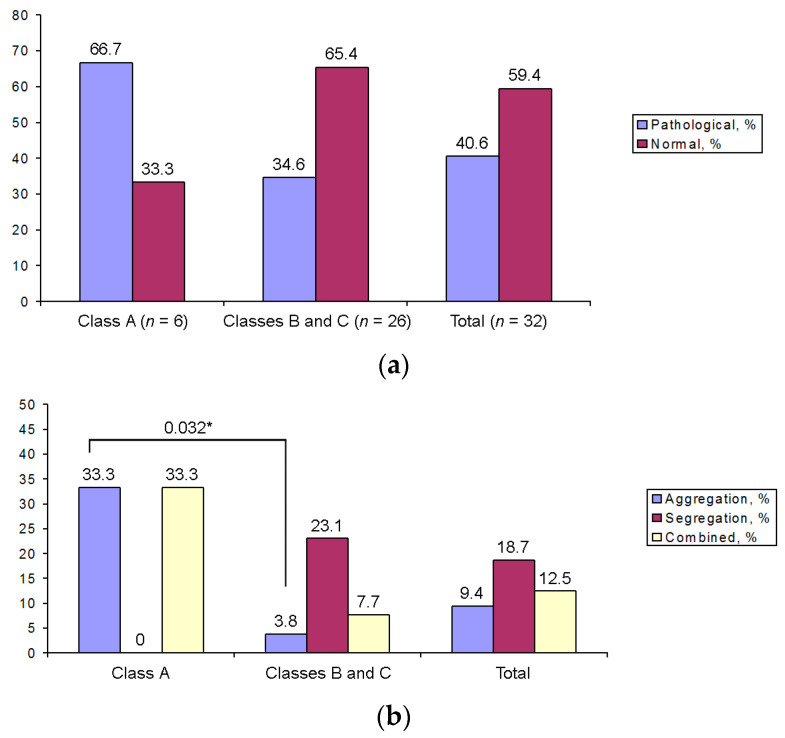
Aneuploidy rates in different classes of GV oocytes (**a**), including the error source (**b**); asterisk indicates significant difference and corresponding *p*-value.

High aneuploidy rates were found in oocytes of all classes (Figure 10a). In Class A oocytes, which have the lowest chromatin aggregation, the frequency of aneuploidy seemed to be slightly higher than in Class B and C oocytes, but these differences are not statistically relevant. As for the source of aneuploidy, segregation errors are the main cause of aneuploidy in Class B and C oocytes, while aggregation errors and a combined cause lead to aneuploidy in Class A oocytes. Statistically significant differences were found between Class A and Class B/C configurations, but only for aneuploidy caused by aggregation errors (Figure 10b).

## 4. Discussion

Our study was devoted to the search for new predictors of oocyte quality, as it is important to increase the percentage of successful outcomes in ARTs [21]. Older reproductive age and a low AMH level are traditionally considered unfavorable anamnestic factors, probably due to a decrease in the number of available oocytes and disturbances in the regulation of the growth and maturation of follicles [34]. In addition, endometriosis reduces the prognosis of successful IVF programs [35] and, as shown in our study, can reduce the quality of oocytes by affecting their maturation during meiotic prophase. At the same time, we have documented that the initial number of antral follicles before the start of the cycle, as well as body mass index and the cause of infertility, are not significant factors. Neither the stimulation protocol chosen for ovarian superovulation nor ovarian responses are predictive of spontaneous activation of GV oocytes. Moreover, the ability of oocytes to resume meiosis does not correlate with the embryonic and clinical outcomes of the forthcoming infertility treatment program.

The possibility of using immature (GV and MI) oocytes in in vitro fertilization (IVF) programs is recurrently discussed [36]. The use of GV oocytes for IVF results in an increase in the average number of available MII oocytes, one additional blastocyst per cycle, and, finally, a higher live birth rate [23,37]. The use of immature oocytes obtained after hormonal stimulation followed by in vitro maturation (IVM) is called the GV rescue [23] and can be considered as an additional assisted reproductive technology (ART) if the number of mature oocytes retrieved during IVF is incomparably less than expected as determined by folliculometry. Successful rescue in vitro maturation (IVM)−ICSI performed shortly after PB extrusion was previously reported, resulting in a successful pregnancy and birth of a healthy baby by a patient who had no mature oocytes at the time of oocyte retrieval [38]. 

One of the few previous studies on human GV oocytes has demonstrated a relationship between follicle diameter, oocyte size, and morphology of the chromatin compartment [14]. Another study [15] showed that GV oocytes obtained in stimulated IVF cycles significantly exceed the size of those oocytes that are not capable of resuming meiosis after IVM cycles. In our study, we used only GV oocytes from dominant follicles (small and medium), which stopped their growth and no longer increased in size. We found no differences in the study groups in terms of follicle and oocyte size, GV position, and ZP thickness. This is consistent with previous works [18,33], which found no relationship between GV position and the quality of human oocytes, despite the fact that such a relationship was shown for mouse oocytes [20].

At the same time, we were able to demonstrate a reliable relationship between the state of the heterochromatin compartment and the ability of GV oocytes to resume meiosis and complete maturation. A low level of chromatin condensation (Class A oocytes) correlates negatively with the ability of the oocyte to resume meiosis, while an intermediate level of chromatin condensation in oocytes containing an incomplete peri-ANu rim (karyosphere) and numerous additional heterochromatin blocks in the nucleoplasm (Class B oocytes) correlates positively [13,14], therefore suggesting that the degree of chromatin condensation in human GV oocytes can be considered as a valuable predictor of spontaneous maturation in stimulated cycles. However, Class C oocytes that exhibit a fully formed karyosphere, when all the chromatin rims the ANu [11], displayed no significant correlation with spontaneous maturation—probably, due to some unconsidered factors. Chromatin was the most dynamic in Class B oocytes cultured in vitro for 6 and 18 h. In Class C oocytes, however, chromatin is less dynamic, which may be linked with the cessation of general transcription activity towards the end of the GV stage [11].

The main cause of pre- and post-implantation arrest of embryo development and spontaneous abortions are aneuploidies resulting from disturbances in the segregation of homologous chromosomes and sister chromatids during maturation of the oocyte after GVBD, at stages MI and MII, respectively [39,40]. It was interesting to examine how the incomplete process of karyosphere formation—chromatin aggregation on the ANu surface—affected the level of pre-segregation and segregation aneuploidies. Using LSI and CEP probes for FISH, we found that GV oocytes with a low level of chromatin condensation exhibit a higher level of aneuploidy upon resumption of meiosis, suggesting that abnormal chromatin aggregation at the GV stage may contribute to the occurrence of aneuploidy. In oocytes with a high level of chromatin condensation, the most common cause of aneuploidy is chromosome segregation errors.

The mechanisms causing aneuploidy at this stage have not yet been studied in detail. There are quantitative data on aneuploidy in oocytes that have undergone spontaneous maturation and subsequent activation, obtained by FISH and regarding different levels of chromosome aggregation, but data on the source of aneuploidy were not presented [22]. The existence of a pre-segregation source of aneuploidy is currently being discussed [41]. An aneuploid set of chromosomes was documented in GV oocytes, accounting for 20% of all cases of aneuploidy [42]. In another study, using microarray comparative genomic hybridization, approximately 15% of GV and MI oocytes exhibited such aneuploidies, the putative cause of which is gonadal mosaicism [43]. In our opinion, disturbances at the stage of chromatin aggregation in GV oocytes, leading to the appearance of aneuploid MI oocytes after GVBD, are also not excluded.

The study of the mechanisms linking chromatin condensation and the incidence of aneuploidy was not within the scope of this study. Nevertheless, we can assume that the condensation of chromatin around the atypical nucleolus and the formation of the karyosphere contribute to the spatial organization of meiotic chromosomes. Impaired formation of the karyosphere, including due to impaired expression of key heterochromatinization proteins, can lead to an increase in the frequency of errors in chromosome behavior during meiotic divisions and, as a result, to the formation of aneuploid oocytes/embryos, as shown, for example, for mice with impaired expression of chromatin remodeling protein ATRX [29].

As for the ANu, it apparently makes a significant contribution to maintaining the integrity of the genome during meiotic divisions, as well as the first divisions of cleavage. However, from our point of view, the ANu plays a role only as a scaffold for chromosomes in their spatial organization. It is difficult to believe that nucleolar transcription plays a significant role in these processes since by the end of the diplotene of female meiosis, the nucleoli turn into transcriptionally inactive homogeneous fibrillar structures—NLBs/ANu [44,45,46,47,48].

Summarizing, the main conclusion of our study is that human GV oocytes that are not capable of successfully completing meiosis display predominantly a low level of chromatin assemblage into a karyosphere. Given that the level of chromatin condensation can be determined in vivo using light microscopy, we believe that this morphological characteristic can be considered a promising criterion for selecting GV oocytes of the best quality in rescue IVM programs.

## Figures and Tables

**Table 1:** Nomenclature for GV oocytes analyzed in the study.

Class	Description	State of the Karyosphere
A	Chromatin is diffuse; only single/scarce heterochromatin clumps are observed in the vicinity of the atypical nucleolus (ANu)	No karyosphere
B	All intermediate pictures between classes A and C; the ANu may be embedded into a chromatin mass, but much condensed chromatin is still distributed throughout the GV; the peri-ANu heterochromatin rim, if in existence, is often incomplete	Incomplete karyosphere in most cases
C	All the chromatin surrounds the ANu	Fully formed compact karyosphere

**Table 2:** DNA probes used for FISH.

Probe Name	Chromosomal Region	Fluorophore	Wavelength (Excitation/Emission, λ_max_)
Vysis LSI 13	13q14.2	Spectrum Red	596/615 nm
Satellite II DNA Vysis CEP 16	16q11.2	Spectrum Aqua	434/481 nm
Alpha Satellite DNA CEP 18	18p11.1–q11.1	Spectrum Blue	405/449 nm
Vysis LSI 21	21q22.13–q22.2	Spectrum Green	499/522 nm
Vysis LSI 22	22q11.2	Spectrum Gold	537/559 nm

**Table 3:** Anamnestic predictors of GV oocyte maturation.

Parameter		Groups of Oocytes			*p*-Value		
		I (*n* = 90)	II (*n* = 14)	III (*n* = 19)	I/III	II/III	I/II
Age, years ^a^		33.0 (29.0–36.0)	31.0 (30.0–33.0)	33.0 (32.0–37.0)	0.394	0.046 *	0.377
FSH, mIU/mL ^a^		6.9 (6.0–9.1)	7.8 (6.3–9.6)	7.1 (6.2–10.3)	0.695	0.760	0.432
AMH, ng/mL ^a^		2.5 (1.6–4.4)	3.5 (1.7–6.1)	1.4 (0.4–2.4)	0.020 *	0.069	0.522
Number of antral follicles ^a^	right ovary	7.0 (5.0–9.0)	6.0 (4.0–8.0)	6.0 (5.0–9.0)	0.683	0.843	0.621
	left ovary	6.0 (4.0–8.0)	7.0 (5.0–8.0)	5.0 (2.0–8.0)	0.264	0.226	0.601
Infertility, % ^b^	right ovary	36.7(33)	64.3 (9)	36.8 (7)	0.988	0.119	0.050
	left ovary	63.3(57)	35.7 (5)	63.2 (12)	0.988	0.119	0.050
PCOS, % ^b^		23.3 (21)	50.0 (7)	36.8 (7)	0.221	0.449	0.036 *
POF, % ^b^		12.2 (11)	21.4 (3)	21.2 (4)	0.310	0.979	0.348
Endometriosis, % ^b^		16.7 (15)	14.3 (2)	36.8 (7)	0.046 *	0.150	0.822
Ovarian resection, % ^b^		18.9 (17)	7.1 (1)	0.0 (0)	0.039 *	0.237	0.279

^a^ Mann–Whitney test; ^b^ Fisher’s χ^2^ test; * significant differences (*p* < 0.05).

**Table 4:** Effect of controlled ovarian stimulation of superovulation on the maturation of GV oocytes.

Parameter		Groups of Oocytes			*p*-Value		
		I (*n* = 90)	II (*n* = 14)	III (*n* = 19)	I/III	II/III	I/II
GnRH agonist, %	long protocol	32.2 (*n* = 29)	50.0 (*n* = 7)	47.4 (*n* = 9)	0.208	0.881	0.193
	short protocol	67.8 (*n* = 61)	50.0 (*n* = 7)	52.6 (*n* = 10)	0.208	0.881	0.193
Trigger hCG, %	manufacturer 1	70.0 (*n* = 63)	71.4 (*n* = 10)	68.4 (*n* = 13)	0.892	0.853	0.913
	manufacturer 2	22.2 (*n* = 20)	21.4 (*n* = 3)	21.1 (*n* = 4)	0.911	0.979	0.947
Trigger hCG, %	manufacturer 1	70.0 (*n* = 63)	71.4 (*n* = 10)	68.4 (*n* = 13)	0.892	0.853	0.913
	manufacturer 2	22.2 (*n* = 20)	21.4 (*n* = 3)	21.1 (*n* = 4)	0.911	0.979	0.947

**Table 5:** Clinical outcomes and maturation of GV oocytes.

Category	Groups of Oocytes			*p*-Value		
	I	II	III	I	II	III
E_2,_ pg/mL *	6522.0 (3804.0–11,680.0)	8031.0 (5964.0–11,255.0)	9161.5 (3461.0–12,708.0)	0.773	0.694	0.233
Number of follicles *	14.0 (10.0–18.0)	15.5 (14.0–16.0)	16.0 (12.0–17.0)	0.478	0.872	0.436
Total number of oocytes *	12.0 (8.0–17.0)	14.0 (12.0–16.0)	14.0 (12.0–16.0)	0.453	0.733	0.562
GV, % *	27.3 (14.3–40.0)	27.2 (16.7–35.7)	28.6 (25.0–35.7)	0.264	0.439	0.966
MII, % *	52.9 (40.0–64.7)	52.8 (35.3–58.3)	42.9 (31.3–58.3)	0.170	0.627	0.627
Good quality embryos D3, % *	50.0 (40.0–80.0)	80.0 (50.0–100.0)	50.0 (40.0–80.0)	0.821	0.163	0.05
Good quality blastocysts D5, % *	42.8 (30.0–63.6)	40.0 (33.3–50.0)	40.0 (33.3–63.1)	0.805	1.000	0.717
CPR, % **	41.1	28.6	47.4	0.616	0.275	0.372

* Mann–Whitney test; ** Fisher’s χ^2^ test.

**Table 6:** Large-scale morphometry of GV oocytes.

Parameter	Groups of Oocytes			*p*-Value		
	I	II	III	I/III	II/III	I/II
D_o_, µm	114.0 (112–116)	116.0 (113–119)	112.0 (108–116)	0.104	0.042 *	0.216
d_o_, µm	108.5 (105–112)	111.0 (106–114)	109.0 (105–112)	0.620	0.461	0.199
S_o_, µm^2^	9671.5 (9324–10,203)	10,117.2 (9676–10,423)	9675.8 (8991–10,116)	0.660	0.106	0.082
D, µm	111.0 (109–114)	113.5 (111–115.5)	111.0 (107–114)	0.582	0.114	0.089
ZP_min_, µm	13.0 (12–16)	14.0 (10–15)	14.0 (12–16)	0.707	0.653	0.803
ZP_max_, µm	18.0 (16–20)	16.5 (15–21)	19.0 (16–20)	0.877	0.843	0.696
S_GV_, µm^2^	699.6 (635–737)	711.6 (624–766)	784.6 (697–840)	0.0001 *	0.065	0.425
D_GV_, µm	29.8 (28.4–30.6)	30.1 (28.2–31.2)	31.6 (29.8–33)	0.0001 *	0.065	0.425

* significant differences (*p* < 0.05); Mann–Whitney test.

**Table 7:** Time intervals of GV oocyte maturation.

Parameter	Groups of Oocytes			*p*-Value		
	I	II	III	I/III	II/III	I/II
T_GVBD_, min	220.5 (106–454)	1478.0 (551–3415)	N/A	N/A	N/A	0.00001 *
T_PB1ex_, min	995.0 (930–1077)	N/A	N/A	N/A	N/A	N/A

* significant difference (*p* < 0.05); N/A, not applicable; Mann–Whitney test.

## Data Availability

Data available on request due to restrictions, e.g., privacy or ethics.

## Supplementary Materials

The following supporting information can be downloaded at: https://www.mdpi.com/article/10.3390/cells12151976/s1, Figure S1: Factors of infertility in the analyzed IVF treatments. Table S1: Average clinical parameters of patients.

## Appendix A

This appendix illustrates some methodical approaches described in Section 2.3 and Section 2.5 (Figure A1 and Figure A2).

## References

[1] Farini D., De Felici M. (2022). The beginning of meiosis in mammalian female germ cells: A never-ending story of intrinsic and extrinsic factors. Int. J. Mol. Sci..

[2] Palmerini M.G., Antonouli S., Macchiarelli G., Cecconi S., Bianchi S., Khalili M.A., Nottola S.A. (2022). Ultrastructural evaluation of the human oocyte at the germinal vesicle stage during the application of assisted reproductive technologies. Cells.

[3] Jiang Y., He Y., Pan X., Wang P., Yuan X., Ma B. (2023). Advances in oocyte maturation in vivo and in vitro in mammals. Int. J. Mol. Sci..

[4] Strączyńska P., Papis K., Morawiec E., Czerwiński M., Gajewski Z., Olejek A., Bednarska-Czerwińska A. (2022). Signaling mechanisms and their regulation during in vivo or in vitro maturation of mammalian oocytes. Reprod. Biol. Endocrinol..

[5] Conti M., Franciosi F. (2018). Acquisition of oocyte competence to develop as an embryo: Integrated nuclear and cytoplasmic events. Hum. Reprod. Update.

[6] Coticchio G., Dal Canto M., Renzini M.M., Guglielmo M.C., Brambillasca F., Turchi D., Novara P.V., Fadini R. (2015). Oocyte maturation: Gamete-somatic cells interactions, meiotic resumption, cytoskeletal dynamics and cytoplasmic reorganization. Hum. Reprod. Update.

[7] Sirait B., Wiweko B., Jusuf A.A., Iftitah D., Muharam R. (2021). Oocyte competence biomarkers associated with oocyte maturation: A review. Front. Cell Dev. Biol..

[8] Ozturk S. (2022). Molecular determinants of the meiotic arrests in mammalian oocytes at different stages of maturation. Cell Cycle.

[9] Bogolyubova I., Bogolyubov D. (2020). Heterochromatin morphodynamics in late oogenesis and early embryogenesis of mammals. Cells.

[10] Fulka J.J., Benc M., Loi P., Langerova A., Fulka H. (2019). Function of atypical mammalian oocyte/zygote nucleoli and its implications for reproductive biology and medicine. Int. J. Dev. Biol..

[11] Parfenov V., Potchukalina G., Dudina L., Kostyuchek D., Gruzova M. (1989). Human antral follicles: Oocyte nucleus and the karyosphere formation (electron microscopic and autoradiographic data). Gamete Res..

[12] Bogolyubov D.S. (2018). Karyosphere (karyosome): A peculiar structure of the oocyte nucleus. Int. Rev. Cell Mol. Biol..

[13] Combelles C.M.H., Cekleniak N.A., Racowsky C., Albertini D.F. (2002). Assessment of nuclear and cytoplasmic maturation in in-vitro matured human oocytes. Hum. Reprod..

[14] Miyara F., Migne C., Dumont-Hassan M., Le Meur A., Cohen-Bacrie P., Aubriot F.-X., Glissant A., Nathan C., Douard S., Stanovici A. (2003). Chromatin configuration and transcriptional control in human and mouse oocytes. Mol. Reprod. Dev..

[15] Sánchez F., Romero S., De Vos M., Verheyen G., Smitz J. (2015). Human cumulus-enclosed germinal vesicle oocytes from early antral follicles reveal heterogeneous cellular and molecular features associated with in vitro maturation capacity. Hum. Reprod..

[16] Mao L., Lou H., Lou Y., Wang N., Jin F. (2014). Behaviour of cytoplasmic organelles and cytoskeleton during oocyte maturation. Reprod. Biomed. Online.

[17] Yamochi T., Hashimoto S., Amo A., Goto H., Yamanaka M., Inoue M., Nakaoka Y., Morimoto Y. (2016). Mitochondrial dynamics and their intracellular traffic in porcine oocytes. Zygote.

[18] Bellone M., Zuccotti M., Redi C.A., Garagna S. (2009). The position of the germinal vesicle and the chromatin organization together provide a marker of the developmental competence of mouse antral oocytes. Reproduction.

[19] Levi M., Ghetler Y., Shulman A., Shalgi R. (2013). Morphological and molecular markers are correlated with maturation-competence of human oocytes. Hum. Reprod..

[20] Almonacid M., Ahmed W.W., Bussonnier M., Mailly P., Betz T., Voituriez R., Gov N.S., Verlhac M.-H. (2015). Active diffusion positions the nucleus in mouse oocytes. Nat. Cell Biol..

[21] ESHRE Special Interest Group of Embryology and Alpha Scientists in Reproductive Medicine (2017). The Vienna consensus: Report of an expert meeting on the development of ART laboratory performance indicators. Reprod. Biomed. Online.

[22] Escrich L., Grau N., Mercader A., Rubio C., Pellicer A., Escribá M.-J. (2011). Spontaneous in vitro maturation and artificial activation of human germinal vesicle oocytes recovered from stimulated cycles. J. Assist. Reprod. Genet..

[23] Escrich L., Galiana Y., Grau N., Insua F., Soler N., Pellicer A., Escribá M. (2018). Do immature and mature sibling oocytes recovered from stimulated cycles have the same reproductive potential?. Reprod. Biomed. Online.

[24] Gremeau A.-S., Andreadis N., Fatum M., Craig J., Turner K., McVeigh E., Child T. (2012). In vitro maturation or in vitro fertilization for women with polycystic ovaries? A case–control study of 194 treatment cycles. Fertil. Steril..

[25] Das M., Son W.-Y., Buckett W., Tulandi T., Holzer H. (2014). In-vitro maturation versus IVF with GnRH antagonist for women with polycystic ovary syndrome: Treatment outcome and rates of ovarian hyperstimulation syndrome. Reprod. Biomed. Online.

[26] Lee J.B., Lee M.G., Lin T., Shin H.Y., Lee J.E., Kang J.W., Jin D.-I. (2019). Effect of oocyte chromatin status in porcine follicles on the embryo development in vitro. Asian-Australas. J. Anim. Biosci..

[27] Monti M., Zanoni M., Calligaro A., Ko M.S.H., Mauri P., Redi C.A. (2013). Developmental arrest and mouse antral not-surrounded nucleolus oocytes. Biol. Reprod..

[28] Monti M., Calligaro A., Behr B., Pera R.R., Redi C.A., Wossidlo M. (2017). Functional topography of the fully grown human oocyte. Eur. J. Histochem..

[29] Baumann C., Viveiros M.M., De La Fuente R. (2010). Loss of maternal ATRX results in centromere instability and aneuploidy in the mammalian oocyte and pre-implantation embryo. PLoS Genet..

[30] Escrich L., Grau N., Meseguer M., Pellicer A., Escribá M.-J. (2010). Morphologic indicators predict the stage of chromatin condensation of human germinal vesicle oocytes recovered from stimulated cycles. Fertil. Steril..

[31] Wada T., Ikegami M., Nagase Y., Yamamoto Y., Matsuda Y., Yonezawa J., Matsuura T. (2014). Maturation rate and subsequent blastocyst development of human oocytes derived from various follicle size at the time of oocyte pick up. Fertil. Steril..

[32] Rienzi L., Balaban B., Ebner T., Mandelbaum J. (2012). The oocyte. Hum. Reprod..

[33] Brunet S., Maro B. (2007). Germinal vesicle position and meiotic maturation in mouse oocyte. Reproduction.

[34] Buratini J., Dellaqua T.T., Dal Canto M., La Marca A., Carone D., Renzini M.M., Webb R. (2022). The putative roles of FSH and AMH in the regulation of oocyte developmental competence: From fertility prognosis to mechanisms underlying age-related subfertility. Hum. Reprod. Update.

[35] Macer M.L. (2012). Endometriosis and infertility: A review of the pathogenesis and treatment of endometriosis-associated infertility. Obstet. Gynecol. Clin. N. Am..

[36] Jie H., Zhao M., Alqawasmeh O.A.M., Chan C.P.S., Lee T.L., Li T., Chan D.Y.L. (2022). In vitro rescue immature oocytes—A literature review. Hum. Fertil..

[37] Martin-Palomino Olid N., García D., Rodríguez A., Vassena R. (2019). Could fertility clinics offer a sizable improvement of live birth rates by maturing post-GVBD oocytes in vitro?. J. Assist. Reprod. Genet..

[38] Otsuki J., Momma Y., Takahashi K., Miyakura S., Nagai Y. (2006). Timed IVM followed by ICSI in a patient with immature ovarian oocytes. Reprod. Biomed. Online.

[39] Holubcová Z., Blayney M., Elder K., Schuh M. (2015). Error-prone chromosome-mediated spindle assembly favors chromosome segregation defects in human oocytes. Science.

[40] Bennabi I., Terret M.-E., Verlhac M.-H. (2016). Meiotic spindle assembly and chromosome segregation in oocytes. J. Cell Biol..

[41] Delhanty J.D.A., SenGupta S.B., Ghevaria H. (2019). How common is germinal mosaicism that leads to premeiotic aneuploidy in the female?. J. Assist. Reprod. Genet..

[42] Costa J., Wilton L. (2000). Aneuploidy of chromosomes 11, 17, 16 and 22 in mature and immature human oocytes. Fertil. Steril..

[43] Daina G., Ramos L., Rius M., Obradors A., Del Rey J., Giralt M., Campillo M., Velilla E., Pujol A., Martinez-Pasarell O. (2014). Non-meiotic chromosome instability in human immature oocytes. Eur. J. Hum. Genet..

[44] Tesařík J., Trávník P., Kopečný V., Kristek F. (1983). Nucleolar transformations in the human oocyte after completion of growth. Gamete Res..

[45] Tesařík J., Kopečny V., Dvořák M., Pilka L., Kurilo L.F. (1984). Human nonovulatory oocyte-cumulus complexes: Ultrastructure, macromolecular synthesis, and developmental potential. Gamete Res..

[46] Zatsepina O.V., Bouniol-Baly C., Amirand C., Debey P. (2000). Functional and molecular reorganization of the nucleolar apparatus in maturing mouse oocytes. Dev. Biol..

[47] Fair T., Hyttel P., Lonergan P., Boland M.P. (2001). Immunolocalization of nucleolar proteins during bovine oocyte growth, meiotic maturation, and fertilization. Biol. Reprod..

[48] Shishova K.V., Lavrentyeva E.A., Dobrucki J.W., Zatsepina O.V. (2015). Nucleolus-like bodies of fully-grown mouse oocytes contain key nucleolar proteins but are impoverished for rRNA. Dev. Biol..

