# Supplementary material for: Chromatin Morphology in Human Germinal Vesicle Oocytes and Their Competence to Mature in Stimulated Cycles
Source: Cells. 2023 Jul 31;12(15):1976. doi: 10.3390/cells12151976 (PMC10416848; doi:10.3390/cells12151976)
Supplement: Supplementary file 1 [file cells-12-01976-s001.zip › cells-2493478-supplementary.pdf]

Supplement 1

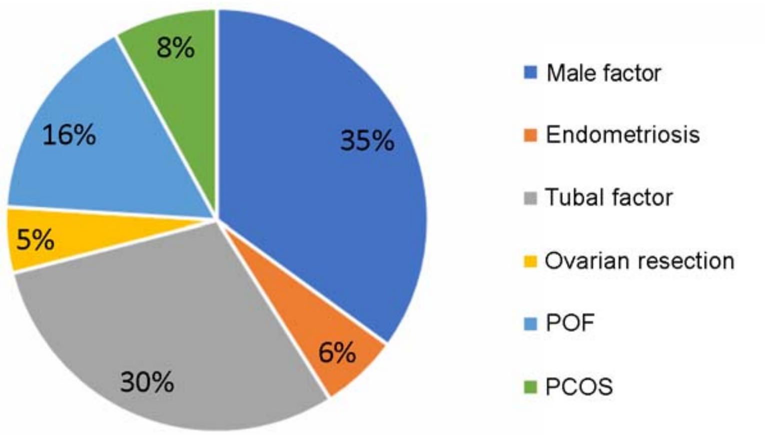

Figure 1S. Factors of infertility in the analyzed IVF treatments.

Table 1S. Average clinical parameters of patients *.

**Parameter**		**Mean ± SD**
Age, years		32.7 ± 4.1
FSH, mIU/mL		7.7 ± 2.6
AMH, ng/mL		3.0 ± 2.1
TSH, μIU/mL		1.9 ± 2.0
Number of antral follicles	right ovary	7.0 ± 3.4
	left ovary	6.2 ± 4.2
BMI, kg/m^2		22.9 ± 3.8

* AMH, anti-Müllerian hormone; BMI, body mass index; FSH, follicle stimulating hormone; TSH, thyroid-stimulating hormone.
